# Effect of Density and Lineage on Dorsal Surface Temperature, Performance, and Carcass Condemnation of Broiler Grillers

**DOI:** 10.3390/ani14152195

**Published:** 2024-07-28

**Authors:** Iara Cristina Marins, Bruno Giacomelli, Bruna Correia, Débora Cristina Olsson, Fabiana Moreira, Juahil Martins de Oliveira Júnior, Ivan Bianchi, Elizabeth Schwegler, Candice Bergmann Tanure, Monike Quirino, Tiago do Prado Paim, Natalia Nogueira Fonseca, Betina Raquel Cunha dos Santos, Vanessa Peripolli

**Affiliations:** 1 Programa de Pós-Graduação em Produção e Sanidade Animal, Instituto Federal Catarinense, Campus Araquari, Araquari 89245-000, SC, Brazil; iaramarins.zoo@gmail.com (I.C.M.); fabiana.moreira@ifc.edu.br (F.M.); ivan.bianchi@ifc.edu.br (I.B.); elizabeth.schwegler@ifc.edu.br (E.S.); quirinomonike@gmail.com (M.Q.); cunhabrs@yahoo.com.br (B.R.C.d.S.); 2 Seara Alimentos Ltda, Itajaí 88305-030, SC, Brazil; giacomelli@vetanco.com.br; 3 Núcleo de Pesquisa, Ensino e Extensão em Produção Animal, Instituto Federal Catarinense, Campus Araquari, Araquari 89245-000, SC, Brazil; b-ru-correia@hotmail.com (B.C.); juahil.oliveira@ifc.edu.br (J.M.d.O.J.); 4 Instituto Federal Catarinense, Campus Concórdia, Concórdia 89703-720, SC, Brazil; debora.olsson@ifc.edu.br; 5 Lohmann Breeders Canada, Brantford, ON N3S 7W4, Canada; c.bergmann@gmail.com; 6 Instituto Federal Goiano, Campus Rio Verde, Rio Verde 75901-970, GO, Brazil; pradopaim@hotmail.com (T.d.P.P.); natalia.nogueira@ifgoiano.edu.br (N.N.F.)

**Keywords:** ambience, chicken, dark house, dermatosis, thermography

## Abstract

**Simple Summary:**

Thermographic monitoring of the back surface of broiler grillers and the environment are accurate indicators of thermal comfort, allowing production to be enhanced and ensuring the quality of the final product. In this scenario, infrared thermography was used to evaluate chickens’ thermophysiological state related to bird density, lineage, age, and time of day, isolated or integrated, on dorsal surface temperature, performance and carcass condemnations of broiler grillers reared in a Dark House system. The main results revealed that the dorsal surface temperature in the broiler grillers was affected by the combined effects of age and time of day, lineage and density, and lineage and age. The increase in the density of broiler grillers did not negatively influence mortality, average daily weight gain, and carcass condemnation. However, partial condemnations and arthritis condemnations were influenced by the lineage.

**Abstract:**

The aims of this study were (i) to evaluate the effect of density, lineage, age, and time of day on dorsal surface temperature and (ii) to evaluate the effect of density and lineage on performance and carcass condemnations in broiler grillers. The evaluations were carried out in barns with the Dark House system, with two densities, 17 and 19 chickens/m^2^ and two lineages, Cobb and Ross. The dorsal surface temperature of the chickens was measured by infrared thermography at 7, 14, 21, 23, 25 and 27 days of age, four times a day. The average daily weight gain, feed conversion, mortality, partial carcass condemnations, as well as those due to arthritis and dermatosis were also evaluated. The highest dorsal surface temperatures were observed in Cobbs housed at a density of 17 chickens/m^2^, and in Ross housed at a density of 19 chickens/m^2^. Cobbs housed at a 17 chickens/m^2^ density showed the lowest feed conversion compared to Ross at the same density. Ross showed higher dorsal surface temperatures when compared to Cobbs at 14, 21, and 27 days. Cobbs showed higher percentages of partial carcass condemnation and arthritis compared to Ross. The higher density of broiler grillers in the Dark House system does not influence the dorsal surface temperature, performance, dermatosis, arthritis, and partial carcass condemnations.

## 1. Introduction

The poultry sector aims to maximize results and reduce production costs at all stages of the production process, thus optimizing investments for better financial returns. For this reason, the density of chickens housed per square meter (m^2^) becomes a relevant factor for production viability. However, the increase in this density contributes to the appearance of skin lesions, which influences carcass quality, impacting the financial return [1]. The quality of the carcass of chickens has become a challenge within the production process, especially for the production of low-body-weight chickens called broiler grillers commonly exported whole to international markets [2].

Skin diseases are frequent causes of chicken carcass condemnation [3,4]. To ensure a carcass without or at acceptable levels of skin lesion, the chickens must be comfortable, with low-stress level, and exposed to an appropriate environment. The Dark House system uses high technology, controlling all ambient factors, including luminosity control [5]. In this system, chickens are raised with controlled lighting from the beginning to the end of the flock, which allows greater control over the stress of the chickens, allowing an increase in bird density without increasing the incidence of dermatosis [6].

Therefore, thermographic monitoring of the body surface and the environment are accurate indicators of thermal comfort, enhancing production and ensuring the final product’s quality [7]. In birds, variations in body surface temperature depend on peripheral blood flow, which indicates their active efforts to regulate core body temperature [8]. In this scenario, infrared thermography has been used to evaluate the thermophysiological state of chickens [7], with the head, wings, back, and feet being the areas commonly evaluated [9]. Measuring the temperature on the back (dorsal) surface of chickens is preferred because this area is less influenced by external factors such as environmental temperature fluctuations or direct sunlight exposure, providing a more accurate reflection of the bird’s core body temperature [8,10]. The lineage used is also an important point to be considered due to its intrinsic characteristics of animal performance, carcass, productivity, bone development, and profitability [11], as well as the feather growth characteristics, structure, and moulting patterns that are important in commercial environments [12]. Therefore, we hypothesized that density, lineage, age and time of day, isolated or integrated, affect dorsal surface temperature, performance and carcass condemnations of broiler grillers. Thus, the aims of this study were (i) to evaluate the effect of density, lineage, age, and time of day on dorsal surface temperature and (ii) to evaluate the effect of density and lineage on the performance and carcass condemnations of broiler grillers.

## 2. Materials and Methods

### 2.1. Experimental Design

The experiment was carried out in the city of Seara (latitude 27°08′58″ south, longitude 52°18′38″ west and altitude of 550 m), located in Santa Catarina state, Brazil, where the climate is mesothermic, humid type (Cfa) according to the Köppen and Geiger [13] classification with an annual mean temperature of 19 °C.

A total of 423,360 female broilers, from both Cobb and Ross lineages were housed in four Dark House barns system. This system uses high technology, controlling all ambient factors, including luminosity control [5]. The chickens were housed by lineage and at two densities, 17 chickens/m^2^ and 19 chickens/m^2^, not exceeding 30 kg/m and observed from housing until slaughter at 28 days of age, being the average age necessary for the chickens to reach an average weight of 1.5 kg targeted by this griller-type broilers of rearing. Three consecutive flocks were evaluated (replications in time) with a mean interval of 15 days between flocks.

Dark House barns measuring 14× 140 m were used, totalling 1960 m^2^ with the same conditions of ambiance, temperature curve, ventilation time, airspeed, static pressure, use of heaters, lighting, equipment adjustment, environmental conditions, and the number of litters. Chickens were raised with controlled lighting from the beginning to the end of the flock. A conventional lighting schedule of 23 h of light and 1 h of darkness was used in the first week, followed by 20 h of light and 4 h of darkness in the following weeks. The luminosity used was 40 Lux in the first week, 25 in the second week, 15 in the third, and 10 in the fourth week. In addition, a standard schedule was used to control temperature, humidity, ventilation, luminosity, cooling, and heating for the three flocks. This schedule considers the installation’s surface in cubic meters, equipment capacity and the needs of the birds for each phase according to the lineages’ guidelines.

Feed and water were provided ad libitum. The chickens received an isoenergetic and isoproteic diet formulated to meet the nutritional requirements of each period.

### 2.2. Performance Evaluation

The number of dead chickens was counted daily from housing to slaughter and used to calculate the mortality rate of the flock, considering the total number of chickens housed.

The feed conversion index was calculated from the volume of feed consumed by the chickens during the evaluation period, which was from housing to slaughter, divided by the weight gain of the flock in the same period.

### 2.3. Evaluation of the Dorsal Surface Temperature of Chickens

**Figure 1:**
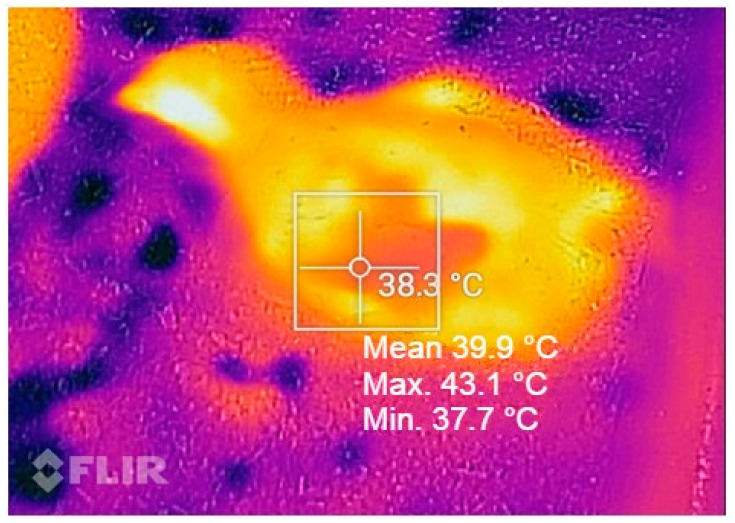
Thermographic image of the dorsal surface temperature of the chickens.

Thermal images were captured on the dorsal (back cape) surface of the chickens, in the upper portion of the wing due to the lower influence of external factors in this body area [8,10] at 7, 14, 21, 23, 25, and 27 days of age as shown in Figure 1. The temperature was randomly performed on three chickens in three areas inside each barn (at the entrance after passing the central door, in the central region where the feeder lines are located at the end of the barn, next to the exhaust fans), at four times: 4:00, 08:00, 14:00 and 20:00 h for each age evaluated.

The sample size used to evaluate the dorsal surface temperature of the chickens was not determined according to the number of chickens in barns but by the expected variation considering the 95% confidence level and expected margin of error of 5% concerning the experimental data according to Cangar et al. [14].

The dorsal temperature of the chickens was measured using a Tg165X infrared thermographic camera (FLIR Systems Inc., Wilsonville, OR, USA). This camera had high precision (±0.1 °C) with an infrared resolution of 320 × 240 pixels and a thermal sensitivity of <0.05 °C at an ambient temperature of 30 °C and an accuracy ±2 °C. The emissivity coefficient (ε) used was 0.94 as suggested by Nääs et al. [10]. An image of each chicken was captured at a distance of 50 cm, using a ruler as a reference, to fill the image in its viewing angles.

View of the back cape region of the broiler where the surface temperature values were extracted.

The square represents the delimitation of the thermal window.

Since the waves of magnetic radiation propagate in a straight line, refraction can occur due to angle distortion generating a change in wavelength. To minimize the error which is almost insignificant for rough surfaces such as the body of the animals, the image was recorded using a 90-degree angle of the chickens’ dorsal surface [9].

Simultaneously, the ambient temperature inside the barns was also measured, based on the readings taken by the controller installed internally.

### 2.4. Evaluation of Carcass Condemnations

The chickens of both lineages were slaughtered following the rules of Brazilian legislation and animal welfare, with a mean live weight of 1500 ± 100 g. In the slaughter line, the chickens were evaluated by the Federal Inspection Service (FIS), where carcass evaluations were carried out, and information on partial condemnations, and condemnations due to arthritis and dermatosis were collected. The evaluation of the carcasses was carried out individually in all chickens during slaughter through a macroscopic visual examination and, when necessary, palpation and cuts were performed.

Condemned chickens due to dermatosis presented skin lesions, and thus could not be packed as whole carcasses, but could be used for other purposes, such as cut-up parts, or processed meat for use in sausages. In condemnations due to arthritis, chickens with hemorrhagic lesions in the joints, which result in the condemnation of the thigh, were considered. The assessment criterion for both evaluations was based on the visual aspect, according to the FIS.

### 2.5. Statistical Analysis

The experimental design was completely randomized with four treatments in a 2 × 2 factorial arrangement, with two chicken densities (17 and 19 chickens/m^2^) and two lineages (Cobb and Ross) with three replications in time (flocks).

Each Dark House barn was considered an experimental unit for evaluations of performance, dermatosis, and arthritis. Each chicken was considered an experimental unit for evaluation of dorsal surface temperature.

The performance, dermatosis, and arthritis data were tested for normality of distribution and homogeneity of residues using Shapiro Wilk and Levene tests, respectively, subjected to Person correlation analysis (PROC CORR) and analyzed using the MIXED procedure in a model that included density and lineage as a fixed effect and flock as a random effect, using the barn as the subject. Interactions between density and lineage were tested. Using Akaike’s information criterion, the variance components (VC) structure was considered the best model for the residual covariance structure. The following statistical model was used:

Yijk = μ + Di + Lj + DLij + Bk + εijk (1)

where Yijk represents dependent variables; μ is the overall mean of the observations; Di is the fixed effect of the density (i = 17 and 19 chickens/m^2^); Lj is the fixed effect of the lineage (j = Cobb and Ross); DLij is the interaction between density and lineage; Bk is the random effect of the flock (k = 1 to 3); and εijk is the random residual experimental error.

The dorsal surface temperature data were tested for normality of distribution and residue homogeneity using Shapiro Wilk and Levene tests, respectively, and analyzed using the MIXED procedure in a model that included density, lineage, age, and time as a fixed effect and flock as a random effect, using the barn as the subject. Interactions between density, lineage, age, and time were tested. Using Akaike’s information criterion, the variance components (VC) structure was considered the best model for the residual covariance structure. The following statistical model was used:

Yijklm = μ + Di + Lj + Ak + Tl + DLij + DAik + DTil + LAjk + TLjk + ATkl + Bm+ εijklm(2)

where Yijklm represents dependent variables; μ is the overall mean of the observations; Di is the fixed effect of the density (i = 17 and 19 chickens/m^2^); Lj is the fixed effect of the lineage (j = Cobb and Ross); Ak is the fixed effect of the age (7, 14, 21, 23, 25, and 27 days); Tl = is the effect of the time (4:00, 08:00, 14:00 and 20:00 h); DLij is the interaction between density and lineage; DAik is the interaction between density and age; DTil is the interaction between density and time; LAjk is the interaction between lineage and age; LTjl is the interaction between lineage and time; ATkl is the interaction between age and time; Bm is the random effect of the flock (k = 1 to 3); and εijkm is the random residual experimental error.

Analyzes were performed using the Statistical Analysis System program (SAS Inst. Inc., Cary, NC, USA, version 9.3), and statistically significant differences were considered when *p* < 0.05.

## 3. Results

There was an interaction between age and time on broiler grillers’ dorsal surface temperature (*p* = 0.0052) (Table 1). The dorsal surface temperatures decreased with the increasing age of the chickens. However, at 21 days, at 20:00, the temperature did not show the expected decline pattern according to the age of the chickens (Table 1).

There was an interaction between density and lineage on dorsal surface temperature (*p* < 0.0001) and feed conversion (*p* = 0.0235) of broiler grillers (Table 2). The density of Cobb chickens at 17 chickens/m^2^ and Ross chickens at 19 chickens/m^2^ had the highest dorsal surface temperatures (Table 2). Cobb chickens at a housed density of 17 chickens/m^2^ had a better feed conversion than Ross chickens at the same density (Table 2).

An interaction between lineage and age significantly influenced the back surface temperature of the broiler grillers (*p* = 0.0003) (Table 3). Ross exhibited higher dorsal surface temperatures than Cobb on days 14, 21, and 27 (Table 3).

There was an effect of the lineage on arthritis condemnation (*p* = 0.0084) and partial carcass condemnation (*p* = 0.0175), where Cobbs had higher percentages for both condemnations compared to Ross (Table 4).

Considering both densities, there was a strong positive correlation between the percentage of dermatosis and partial carcass condemnation. In addition, the percentage of arthritis showed a moderate positive correlation with the mortality rate in chickens (Table 5). Considering the density of 19 chickens/m², there was a strong positive correlation between feed conversion and mortality, between arthritis and feed conversion, and between dermatosis and partial condemnations (Table 5).

## 4. Discussion

The Dark House system has greater potential to produce broilers, presenting superior performance when compared to conventional systems [5]. Barns that use the Dark House system typically have a higher density of broiler chickens, resulting in more contact between chickens [15]. At high densities, chickens need greater heat dissipation, adopting strategies such as opening their wings and ruffling feathers, generating greater movement to maintain higher contact with the air, and ensuring faster cooling, predisposing to a significant increase in carcass condemnations. The Dark House system has reduced condemnation rates for dermatosis caused by scratches [15]. This is due to the high control of the ambient conditions that ensure thermal comfort to the chickens and the greater ease of handling in the barns, especially at the time of catching by controlling the luminosity [15].

Carcass condemnations are significant in the poultry sector, and factors that may contribute to the increase in these condemnations should be minimized. In the present study, the increase in the density of broilers did not cause an increase in mortality and partial carcass condemnations, as well as arthritis and dermatosis, corroborating with previous studies [16,17,18,19,20]. However, there was an interaction between density and lineage on dorsal surface temperature and feed conversion, which can be explained by the performance curve of each lineage, where broilers from commercial lineages presented different genetic potentials due to selection pressure applied to the characteristics of economic interest, such as weight gain, feed conversion, and resistance, resulting in differences in the lineage’s profile, making each lineage present a specific performance and behave differently when subjected to different densities [21].

Among commercial lineages, Cobb presents better productive performance, among commercial lineages, due to better weight gain, as it has excellent feed conversion, high muscle deposition capacity, greater rusticity, and resistance to handling, regardless of density [22]. Marcato et al. [23] also observed that Cobb had a higher growth rate and nutrient deposition, reaching a higher slaughter weight when compared to Ross.

In the present study, the lineages did not affect the average daily weight gain, consistent with Lara et al.’s [22] findings of Ross showing similar productivity to Cobb. However, the former has lower initial growth, with a compensatory gain after 21 days of age, obtaining high final weight gain, generally superior to that of Cobb. For the same authors, Ross showed better viability, with a lower percentage of mortality and better use at slaughter due to lower carcass condemnations, which was also observed in the present study.

The dorsal surface temperature was higher in chickens reared at lower density, which demonstrates a greater ability to control the environment even at high densities in the Dark House system. This system allows the use a higher density of chickens per square meter in the barns due to the use of technology to control the environment, allowing lower percentages of dermatosis, better feed conversion, and greater average daily weight gain [15]. Thus, the ambient control and the technology of the Dark House system used in the present study allowed maintenance of the mortality rate, the average daily weight gain, and the carcass quality of the chickens even when raised in higher density.

In the present study, Cobb presented higher percentages of partial condemnations and arthritis. According to Lara et al. [22], Cobb has a higher capacity for muscle deposition and better feed conversion, however, this rapidly developing musculature can make the skin more exposed and susceptible to scratches, resulting in higher percentages of dermatitis, pododermatitis, and inflammation.

In commercial lineages, one of the determining factors for carcass quality is the rate of feathering. The earlier the onset of feathering, the lower the percentage of dermatosis-related carcass condemnation, since the feathers act as a protective barrier and help avoid the appearance of skin lesions in commercial broilers raised at high densities. The feathering process involves complex physiological mechanisms, influenced by nutritional, hormonal, genetic, and environmental factors, as well as by the interaction between them [24]. Some broiler lineages with slower feathering rates tend to have a higher incidence of skin lesions [12]. Since Cobb has a faster feathering rate when compared to Ross [12], the former was expected to have lower percentages of partial carcass condemnations. However, the early feathering trait alone was not enough to minimize partial carcass condemnations, and the fast-growing trait should be considered when choosing the ideal lineage to produce broilers in a Dark House system.

Very high densities compromise chicken welfare, and performance, increasing surface temperatures [25]. Therefore, it is crucial to know the factors that influence the surface temperature, and the thermoregulation mechanisms of chickens to minimize the impacts of increased density [26]. Infrared thermography is a noninvasive remote sensing method used to measure changes in heat transfer and blood flow in humans and animals by detecting small variations in body temperature, providing valuable information [10,27]. Abudabos et al. [25] reported that birds housed in medium and high densities exhibited higher body, head, neck, wing, and shin temperatures compared to those in lower densities. The authors concluded that the increased density negatively impacted the welfare and performance of the birds. Therefore, knowing the surface temperature allows adjustment of the environmental temperature according to the needs of the chickens. In the present study, the dorsal surface temperature of the chickens was influenced by the integrative effect between age and time, between lineage and density, and between lineage and age.

It is important to consider that the surface temperature varies in response to environmental and physiological conditions [10]. The feathered parts of the body have a lower response to environmental temperature, as opposed to the parts with no feathers, thus highlighting the impact of the environmental temperature [8]. Thus, assessments performed on the dorsal area of the chicken represent a slower response to the environment [8,10].

The decrease in body temperature with increasing age should be gradual and points to the comfort condition of the chickens [14]. In the present study, there was a gradual decrease in the dorsal surface temperatures as the chickens got older, stabilizing at 23 days of age at all times of the day. However, at 21 days, at 20:00, the temperature did not show the same temperature decline pattern considered normal according to the age of the chickens.

At the beginning of life, chickens have higher difficulty in thermal regulation, being greatly influenced by the environment temperature. The high temperatures in the first weeks of life, as observed in the present study, may be related to environmental factors, which directly reflect on the surface temperature of young chickens. In addition, environmental humidity can influence the surface temperature of one-week-old birds, redistributing heat, and generating changes between body and surface temperature [28].

The gradual decrease in temperature up to 21 days coincided with the period of development of the thermoregulatory system and with the feathering of the chickens [29]. Thus, it is recommended to use heaters for at least 21 days, even in summer, because only after this period will the chickens have a fully developed thermoregulatory system [30].

Considering the evaluation of both densities, a moderately positive correlation was observed between arthritis and mortality. As for the 19 chickens/m² density, a strong positive correlation was observed between feed conversion and arthritis. This is because arthritis causes pain and discomfort to chickens, decreasing their performance, which worsens feed conversion [20,21,22,23,24,25,26,27,28,29,30,31]. In addition, arthritis can be fatal for chickens, which explains the positive correlation with mortality.

Thermography can be utilized to assess temperature changes, providing valuable insights into housing conditions and management practices in poultry farming. Furthermore, studying thermoregulatory patterns in chickens through thermography could contribute to genetic selection programs to breed more heat-tolerant and resilient poultry lineages.

## 5. Conclusions

The dorsal surface temperature of broiler grillers in a Dark House system is influenced by the integrative effect of age and time of day, lineage and density, and lineage and age.

The increase in the density of broiler grillers does not negatively influence mortality, average daily weight gain, and carcass condemnation. However, partial condemnations and arthritis condemnations are influenced by lineage.

## Figures and Tables

**Table 1:** Effect of the interaction between age and time on dorsal surface temperature (°C) of broiler grillers.

Age, Days	Time, hh:mm				Mean	SEM
	04:00	08:00	14:00	20:00		
7	40.20 ^Aa^	40.04 ^Aa^	40.67 ^Aa^	40.27 ^Aa^	40.29	0.4523
14	38.26 ^Ba^	38.02 ^Ba^	38.82 ^Ba^	38.39 ^Ba^	38.37	0.4523
21	37.77 ^Bb^	38.19 ^Bb^	39.37 ^Ba^	39.40 ^Aa^	38.68	0.4520
23	36.48 ^Cb^	35.97 ^Cb^	38.37 ^Ba^	38.22 ^Ba^	37.26	0.4516
25	35.55 ^Ca^	35.86 ^Ca^	35.48 ^Ca^	36.01 ^Ca^	35.72	0.4520
27	35.57 ^Ca^	35.98 ^Ca^	35.47 ^Ca^	36.10 ^Ca^	35.78	0.4558
Mean	37.30	37.34	38.03	38.06		
SEM	0.4400	0.4398	0.4401	0.4399		
*p*-value						
Age × time	0.0052					

*p*-value: probability; SEM: standard error of the mean; ^A,B,C^ Different uppercase letters in columns and ^a,b^ different lowercase letters in rows differ by Tukey test at 5% (*p* < 0.05).

**Table 2:** Effect of the interaction between density and lineage on dorsal surface temperature (°C) and feed conversion of broiler grillers.

Lineage	Density		Mean	SEM
	17 Chickens/m^2^	19 Chickens/m^2^		
Back temperature				
Cobb	39.56 ^Aa^	35.73 ^Bb^	37.65	0.7254
Ross	37.84 ^Ba^	38.94 ^Aa^	37.94	0.7270
Mean	38.70	36.88		
SEM	0.7376	0.7297		
*p*-value				
Density × lineage	<0.0001			
Feed conversion				
Cobb	1.35 ^Ba^	1.45 ^Aa^	1.41	0.0195
Ross	1.47 ^Aa^	1.38 ^Aa^	1.42	0.0195
Mean	1.41	1.42		
SEM	0.0201	0.0201		
*p*-value				
Density × lineage	0.0235			

*p*-value: probability; SEM: standard error of the mean; ^A,B^ Different uppercase letters in columns and ^a,b^ different lowercase letters in rows differ by Tukey test at 5% (*p* < 0.05).

**Table 3:** Effect of the interaction between lineage and age on dorsal surface temperature (°C) of broiler grillers.

Age, Days	Lineage		Mean	SEM
	Cobb	Ross		
7	40.20 ^Aa^	40.62 ^Aa^	40.41	0.7397
14	38.03 ^BCb^	38.94 ^Ba^	38.48	0.7397
21	38.39 ^Bb^	39.20 ^Ba^	38.79	0.7395
23	37.70 ^Ca^	36.94 ^Ca^	37.32	0.7392
25	36.13 ^Da^	35.57 ^Ca^	35.85	0.7395
27	35.43 ^Db^	36.36 ^Da^	37.90	0.7421
Mean	37.65	37.94		
SEM	0.7254	0.7270		
*p*-value				
Lineage × age	0.0003			

*p*-value: probability; SEM: standard error of the mean; ^A,B,C,D^ Different uppercase letters in columns and ^a,b^ different lowercase letters in rows differ by Tukey test at 5% (*p* < 0.05).

**Table 4:** Effect of density and lineage on performance variables, dermatosis, arthritis, and partial condemnation of broiler grillers.

Trait	Density		Lineage		Mean	SEM	*p*-Value		
	17 Chickens/m^2^	19 Chickens/m^2^	Cobb	Ross			Density	Lineage	Density × Lineage
Mortality, n	4.97	3.92	5.29	3.60	4.44	0.5498	0.3874	0.1920	0.9491
ADG, g/day	52.15	51.84	51.60	52.40	52.00	0.5846	0.8279	0.5801	0.9328
Dermatosis, %	2.08	2.64	3.10	1.62	1.98	0.2815	0.5698	0.1716	0.5115
Arthritis, %	1.75	1.15	2.20 ^a^	0.69 ^b^	1.43	0.2894	0.2154	0.0084	0.2948
Partial condemnations, %	4.16	4.32	5.59 ^a^	2.89 ^b^	4.24	0.5030	0.8319	0.0175	0.9365

*p*-value: probability; SEM: standard error of the mean; ADG: average daily weight gain; ^a,b^ Different lowercase letters in rows differ by Tukey test at 5% (*p* < 0.05).

**Table 5:** Correlation between performance variables, dermatosis, arthritis, and partial condemnation of broiler grillers.

	ADG	FC	Dermatosis	Arthritis	PC
General					
Mortality	0.03521	−0.31205	−0.01326	**0.66733**	0.28105
ADG	-	−0.22276	0.09458	−0.06804	0.05852
FC	−0.22276	-	0.01022	−0.22737	−0.10804
Dermatosis	0.09458	0.01022	-	−0.04805	0.84695
Arthritis	0.66733	−0.06804	−0.22727	-	0.46889
PC	0.05852	−0.10804	**0.84695**	0.46889	-
17 chickens/m^2^					
Mortality	0.41428	−0.80861	0.00198	0.55180	0.29707
ADG	-	−0.00056	0.04911	0.05201	0.00718
FC	−0.00056	-	−0.21792	−0.77565	−0.62271
Dermatosis	0.04911	−0.21792	**-**	0.12324	0.76456
Arthritis	0.05201	−0.77565	0.12324	-	0.72912
PC	0.00718	−0.62271	0.76456	0.72912	-
19 chickens/m^2^					
Mortality	−0.68482	**0.95953**	0.09813	0.86053	0.37988
ADG	-	−0.52962	0.15082	−0.30487	0.10784
FC	−0.52962	-	0.19722	**0.84843**	0.51176
Dermatosis	0.15082	0.19722	**-**	−0.07926	**0.91839**
Arthritis	−0.30487	**0.84843**	−0.07926	-	0.28611
PC	0.10784	0.51176	**0.91839**	0.28611	-

ADG: average daily weight gain; FC: feed conversion; and PC: partial condemnation. Bold numbers are statistically significant at 5% (*p* < 0.05).

## Data Availability

All data generated and analyzed during the current study are available from the corresponding author upon reasonable request.
